# Phage Therapy: Eco-Physiological Pharmacology

**DOI:** 10.1155/2014/581639

**Published:** 2014-05-20

**Authors:** Stephen T. Abedon

**Affiliations:** Department of Microbiology, The Ohio State University, Mansfield, OH 44906, USA

## Abstract

Bacterial virus use as antibacterial agents, in the guise of what is commonly known as phage therapy, is an inherently physiological, ecological, and also pharmacological process. Physiologically we can consider metabolic properties of phage infections of bacteria and variation in those properties as a function of preexisting bacterial states. In addition, there are patient responses to pathogenesis, patient responses to phage infections of pathogens, and also patient responses to phage virions alone. Ecologically, we can consider phage propagation, densities, distribution (within bodies), impact on body-associated microbiota (as ecological communities), and modification of the functioning of body “ecosystems” more generally. These ecological and physiological components in many ways represent different perspectives on otherwise equivalent phenomena. Comparable to drugs, one also can view phages during phage therapy in pharmacological terms. The relatively unique status of phages within the context of phage therapy as essentially replicating antimicrobials can therefore result in a confluence of perspectives, many of which can be useful towards gaining a better mechanistic appreciation of phage therapy, as I consider here. Pharmacology more generally may be viewed as a discipline that lies at an interface between organism-associated phenomena, as considered by physiology, and environmental interactions as considered by ecology.

## 1. Introduction

*To understand the response of organisms to their environments one needs to understand as thoroughly and rigorously as possible all pieces of the problem. The environment must be known correctly… The organism must be known correctly… including all the functional relationships…—David M. Gates, p. 343, as quoted in Tracy and Turner [1]*

*…research areas at the borderline between microbiology, ecology and physiology are key. Brüssow and Kutter [2]*

The biological sciences, for the past two decades and more, have been pushing exploration of genotype to some approximation of a limit, where today genotype information on organisms, that is, DNA sequence, literally can be obtained faster than it can be studied [3], or even more easily than it can be permanently stored [4]. While genotype information is clearly important towards understanding organism diversity, prevalence, and evolution, it nonetheless provides only as much insight into organism functioning as previous phenotypic characterization has made possible [5], particularly in terms of genotype-phenotype maps [6] along with what can be described as “comparative phenomics” [7, 8]. Characterizing phenotype, despite substantial technological improvement especially in molecular tools, nevertheless remains a tedious endeavor; for example, [9]. Notwithstanding vast improvements in DNA sequencing technologies, obtaining high quality phenotype information therefore continues to represent both a primary challenge and primary goal of biology.

**Figure 1:**
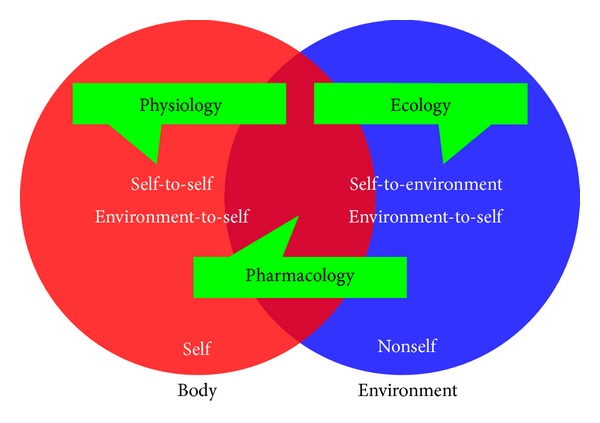
Connections between physiology, ecology, and pharmacology. At best the distinction between an organism's physiology and its overall ecology can be ambiguous, though with body-pharmaceutical interactions representing one aspect of their interface. Such chemicals specifically can be viewed as abiotic components of an organism's environment, ones that have made contact with an organism's tissue, as can also toxins. Shown are body interactions not just with self (as body) but with nonself as well (as environment), with emphasis in physiology on the impact of these interactions on the functioning of self. Ecology also is the study of interactions between self and nonself, but with the consequences of such interactions considered with emphasis on both self, as body, and nonself, as environment. Pharmacology too is the study of interactions between self and nonself (particularly “environment-to-self”), though as with physiology there is an emphasis on impact on self (again, as body).

**Figure 2:**
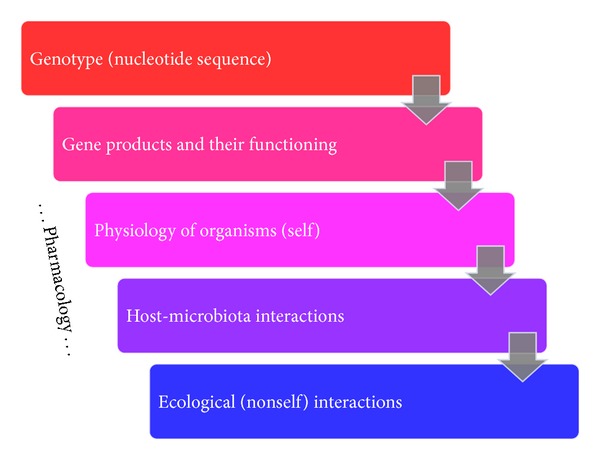
The realm of pharmacology includes an organism's gene products and their functioning, the overall physiology of an organism, the interactions that occur between a host organism and their associated microbiota, and even aspects of an organism's ecology. The realm of pharmacology. Pharmaceuticals interact with individual body molecules, including gene products as well as products of enzyme-mediated catalysis. The goal with pharmacology, in turn, is a modification of the physiology and particularly the pathophysiology of treated organisms. Organisms themselves generally consist of more than just the products of their own genomes but also the products of their associated microbial symbionts [221]. An important component of pharmacology therefore is the interaction of pharmaceuticals with this microflora. Not shown is the impact of drugs that serve either as mutagens or as nucleic acid damaging agents, which can affect genotype as well.

Consideration of individual molecules, as studied in relative isolation, can represent the less complex of tasks in terms of phenotypic characterization. When we consider, however, how these molecular characteristics can determine the processes of organism functioning, thereby defining their physiology, that biology becomes particularly complex. This complexity then acquires an additional dimension when the interaction of organism physiologies with environments is considered, which is the province of ecology. Existing at an interface between ecology and physiology is the interplay between organisms and the various species with which they share symbiotic relationships. Symbioses, specifically, are intermediate to the functioning of an organism's body, on the one hand—such as in terms of development of immunity [10], animal development more generally [11], or even behavior [12] and thus generating what can be described as “supraorganisms” or “metaorganisms” [13, 14]—and a body's environmental interactions on the other. Lastly, we can consider artificial means by which an organism's physiology may be manipulated, as effected, for example, in the course of medical treatments, such as those that involve pharmaceuticals. Pharmacology, that is to say, is an inherently ecological endeavor as pharmaceuticals can be viewed as non-self-components of an organism's environment (Figure 1). At the same time, however, the primary goal of pharmacology is one of patient physiological manipulation (Figure 2).

Among pharmaceuticals are antagonists to bacteria and antibacterials generally can be physical, chemical, or, arguably (as pharmaceuticals), even biological. Physical antibacterials include extremes in pH, temperature, moisture levels, and also various forms of radiation. Chemical antibacterials include disinfecting as well as sterilizing agents. An important category of chemical antibacterials are those that can be applied directly to living tissues, which includes antiseptics, antibiotics, and various synthetic antibacterial drugs. Crucial for the functioning of the latter is what is known as selective toxicity [15], that is, the potential to do harm to target microorganisms while simultaneously avoiding damaging host tissues.

Bacteria can produce antibacterial agents that are too complex to be described as antibiotics, or at least as small-molecule antibiotics. These agents include bacteriocins [16] as well as bacteriophages. Because of the tendency of larger bacteria-produced antibacterial agents to be highly specific in terms of what bacteria or indeed organisms they affect—particularly such agents as phages along with colicin- and pyocin-like antibacterials [17, 18]—they often will display substantial selectivity in their toxicity, which can be helpful towards safeguarding beneficial normal microbiota [16]. These agents also have the utility of being somewhat easily discovered.

In this review, I explore the association that exists between physiology, ecology, and pharmacology, especially in the course of treatment of infectious disease. I focus in particular on the biocontrol of bacterial infections using pathogen-specific bacterial viruses, that is, the nearly one-hundred-year-old antibacterial technique known as phage therapy [19–23]. For reasons of limitations of space and also to avoid excessive repetition with other publications, in a number of places I point the reader to other reviews rather than rereviewing especially earlier material. I examine in particular aspects of the pharmacology of phage therapy that inherently are found at an interface between physiology and ecology.

I begin with a brief history of phages and phage therapy and then provide an introduction to the biology of phages and the concept of phage physiology. This is followed by further introduction to phage therapy as well as phage therapy pharmacology and related issues of phage ecology. Presented next, and making up the bulk of the review, are considerations of ecology, physiology, and pharmacology as found within the context of phage therapy. This includes application of these concepts towards addressing the role of phage virions in phage therapy, including in terms of phage-body interactions. Next considered are phage-bacterial interactions. Related to this category are phage interactions with bacteria that are already phage infected along with interactions between patient bodies and phage-infected bacteria. I then conclude with a more historical consideration of the development of what I describe, here, as the eco-physiological pharmacology of phage therapy.

## 2. History of Phage Therapy

The practice of phage therapy began nearly with the discovery of phages themselves. While a number of authors have suggested that the first evidence of the existence of phages dates back to the late 19th century [24], in fact the generally agreed upon dates of independent discovery [25] are 1915 and 1917 by Twort [26] (see [27] for a recent, open-access republication) and d'Hérelle [28], respectively (for translations of the latter, see [29–31]). d'Hérelle was particularly instrumental as an early student of phages, providing us not only with their name (originally as “un bactériophage obligatoire”) but also observation of their replication in broth and formation of plaques, as well as the publication of the first phage-emphasizing monograph [32].

The first use of phages as antibacterial agents proceeded relatively soon after their discovery, with the first phage therapy publication appearing in 1921 [33]. Approximately over the same period, d'Hérelle [34] was observing a role for naturally occurring bacteriophages in the control of bacterial disease (pp. 181 and 184): “The disease is only definitely overcome at a time when the virulence of the bacteriophage is sufficiently high to dominate the resistance of the bacterium.” “In all cases the fluctuations in the virulence, as well as the fluctuations in the resistance of the bacteria, parallel the state of the patient, and the onset of improvement coincides with the moment when the virulence of the bacteriophage dominates clearly the resistance of the bacterium.”

As outlined in Abedon et al. [20] (but see also Summers [19, 35]), during the 1920s as well as during a portion of the 1930s, there existed substantial enthusiasm for phage therapy among numerous researchers. This enthusiasm, however, was not underlain by any more than superficial understanding of just what phages represented, for example bacterial viruses versus some sort of less dynamic bacterial product. The results were apparently impressive successes in the use of phages to treat bacterial disease (e.g., see [36]) but also sufficient failures in combination with seemingly excessive claims made by proponents that a backlash commenced, starting in earnest in 1934 [37–39] and continuing into the 1940s. What followed was a relative dearth of phage therapy practice, particularly in English-speaking countries, that only began to turn around starting in the 1980s with the work of Smith and Huggins of the United Kingdom [40–42] as well as that documented by Slopek and colleagues in Poland [43].

As also described in some detail in Abedon et al. [20], in other parts of the world—most notably the U.S.S.R., and particularly Georgia, but also Poland and France—the practice of phage therapy remained vibrant even as it faded in English-speaking nations. As the problem of antibiotic resistance became more apparent during the 1990s, however, numerous individuals as well as companies turned both to phage therapy and those institutions, most notably in the now independent former Soviet republic of Georgia, that still routinely practiced phage therapy. The result has been a growing interest in the potential to use phages as antibacterial agents within the context of medicine as well as veterinary medicine, agriculture, and other circumstances, for example, [44–46]. A utility for greater appreciation of the pharmacology of phage use within the context of phage therapy and associated issues of phage ecology can be found starting in the 1990s and into the early 2000s in publications especially by Levin and Bull [47–49] and then Payne and Jansen [50–52].

## 3. Phages and Phage Physiology

**Figure 3:**
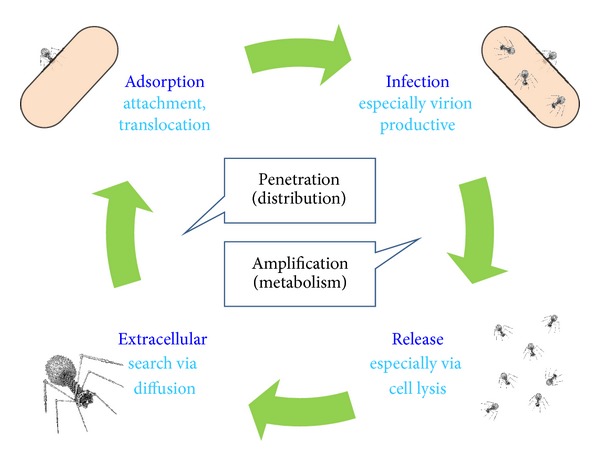
Life cycle of an obligately lytic bacteriophage. As this is a cycle, the “beginning” is arbitrary. A successful infection nonetheless progresses through adsorption, infection, release (here via lysis), and a period of extracellular “search” for new bacteria to infect. Deviations from this life cycle can include inactivation during the extracellular stage, a failure to successfully adsorb, and various forms of phage inactivation that can occur during infection, including as explicitly mediated by bacterial cells [17, 164]. Though lytic phages are released via lysis, other phages exist, most notably filamentous phages such as phage M13, that instead are released from infected bacteria chronically. Generally such nonlytic phages are not used for phage therapy. Another variation on the phage life cycle is lysogenic cycles, which are nonvirion productive extensions of the infection stage. Only temperate, particularly* not *obligately lytic phages display lysogenic cycles, and temperate phages typically also are not among the first choice for phage therapy purposes [222]. Shown too, in the middle, is reference to pharmacological aspects of phage infections. Particularly these are distribution throughout body tissues that can occur while in the free phage state (a.k.a., phage penetration to target bacteria) along with amplification of phage numbers* in situ* as can occur as a consequence of phage infection of bacteria, which is a component of what pharmacokinetically is known as metabolism.

The life cycle of phages can be distinguished into four basic steps (Figure 3). First is an extracellular stage during which the virion capsid protects the phage genome such as from nucleases [53, 54]. This is proceeded as well as preceded by an infection stage during which a majority of phage physiological aspects are observed [55, 56]. Infection ends with release, usually via phage-induced bacterial lysis [57], thus initiating the extracellular phase. The extracellular phase ends and infection begins in the course of what variously is described as attachment, adsorption, uptake, penetration, ejection, injection, and/or translocation [58]. This step in yet other words is the irreversible association of a virion particle with the surface of a bacterial cell along with subsequent steps that result in a phage genome becoming suspended within a bacterium's cytoplasm.

It is the specificity particularly of the attachment step that contributes to the relative safety of phage therapy [59–64], since phages for the most part are unable to deliver cytotoxic activity to cells without first irreversibly attaching to them. In addition, and importantly, the cytotoxic agents that phages deliver to target bacteria [65] tend to be either somewhat specific to bacteria or otherwise functional only following phage virion-specific delivery to the cytoplasms especially of target bacteria [66]. In terms of drug discovery generally [67] (p. 732): “It is likely that evolutionary forces select against scaffolds that cause a high degree of nonspecific interactions with many biological molecules. In a sense, natural products have been field-tested by evolution.”

Successful phage infections involve biosynthesis along with various forms of physiological modification of the infected bacterium; for example, see Calendar and Abedon [68] for overview of details associated with numerous phage types. With larger-genomed phages as favored for phage therapy, which collectively are known as tailed phages [69, 70], the number of genes and physiological steps involved to produce virions can be substantial, up to well over one hundred phage genes, such as the approximately 300 gene products encoded by phage T4 [71]. Little is understood, however, about the impact of subtle physiological details on key endpoints to productive phage infections. The connection, for example, between specific aspects of phage infection physiology and how many phage virions are produced (burst size), or preinfection bacterial physiology and how long it takes to produce those virions (latent period), generally are not well appreciated, except in cases where changes have the effect of substantially reducing phage productivity or modifying periods of infection. Burst size as well as latent period nevertheless can vary between host types as well as growth conditions, implying variation as a consequence of host physiology [56]. A classic study in this regard is that of Hadas et al. [72], which looked the impact of host physiology on these and other phage T4 growth parameters.

Only a subset of bacterial strains tend to be affected physiologically by any one phage type [17]. This somewhat narrow phage host range is important in terms of the safety of phages as antibacterials. It also can be limiting in terms of the potential for phages to impact specific bacterial targets during phages therapy [73, 74], though alternatively so-called superphages exist that possess what for phages are relatively broad spectra of activity, such that potential hosts include a majority of strains making up a single bacterial species [75].

## 4. Phage Therapy and Pharmacology

Phage therapy is a form of biological control, or biocontrol, in this case as mediated by microorganisms [76]. The term biocontrol may be used to describe more food- or environment-oriented treatments. When phages are used as alternatives to antibacterial* drugs* in medicine or veterinary practice [77], however, then this is what can be described specifically as phage therapy [78]. While in principle all bacteria can be impacted by phages, in practice it is especially gastrointestinal afflictions, localized infections, and otherwise chronic infections that are treated within a phage therapy context. For overviews of phage treatment particularly of humans, see [20, 21, 46, 59]. See as well a 2010 volume edited by Sabour and Griffiths [79] that covers especially phage-mediated biocontrol of bacteria, Abedon [80] from the same year for an edited volume reviewing various aspects of phage therapy along with phage-mediated biocontrol of bacteria, a 2012 volume partially covering phage therapy and biocontrol edited by Hyman and Abedon [81], and a fourth edited volume dedicated to phage therapy and phage-mediated biocontrol edited by Borysowski et al. that will soon be published [82]. For a recent article discussing “the limitations on the wider therapeutic use of phage,” see [83] and see also [84]. The phage therapy field nevertheless remains relatively small (Table 1), with approximately 30 equivalent papers published in 2012 (Table 2).

The actual practice of phage therapy is fairly straightforward. One or more phage types that are either thought to be effective against target bacteria or that have been shown to be effective following laboratory testing are administered in some manner to a patient. Ideally these phages can reach and then disrupt target bacteria. Disruption can be accomplished by killing bacteria, clearing biofilms [85], and perhaps also by increasing bacterial susceptibility to existing host immunity. Indeed, it has long been postulated that phages may play roles as components of a body's normal microbiota as a natural defense against bacteria [86]. From d'Hérelle [34] (p. 171), for example, “If the bacteriophage is an agent of immunity, it will not appear only at the exact moment when it is most needed. It should be a normal inhabitant of the intestine.” See also a review of this subject endogenous phages and their potential role in pathogen resistance by Górski and Weber-Dabrowska [87] and more recently as postulated by Barr et al. [88] with regard to specific association by phages with animal mucus.

Considerations of treatment choice, routes of treatment administration, treatment success, and avoidance of side effects are standard pharmacological considerations for any drug. They represent issues of drug spectrum of activity (as well as other pretreatment considerations such as drug cost), drug pharmacokinetics, drug primary pharmacodynamics, and drug secondary pharmacodynamics, respectively. Pharmacokinetics specifically considers the body's impact on a drug whereas pharmacodynamics instead is a description of a drug's impact on the body. Pharmacokinetics also is a description of a drug's ability to reach target tissues in sufficient densities to be effective while pharmacodynamics is a description of what a drug is capable of accomplishing, both positively and negatively, once those densities have been reached. It is traditional also to differentiate pharmacokinetics into what are known as absorption, distribution, metabolism, and excretion. These, respectively, represent drug uptake principally into the blood, drug movement to other body tissues (and particularly out of the blood), drug modification (usually but not exclusively towards inactivation, e.g., [89, 90]), and drug physical removal from the body.

**Figure 4:**
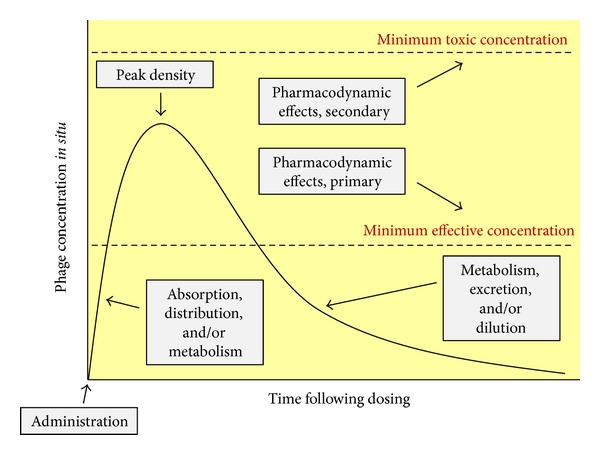
Basics of phage therapy pharmacology. Absorption and distribution can have the effect of increasing antibacterial concentrations within the vicinity of target bacteria, though also they have a diluting effect on dosages. Phage infection too can increase phage numbers within the vicinity of target bacteria, which I have indicated as being an aspect of metabolism and which more generally is a description of the chemical modification of a drug. Together these pharmacokinetic mechanisms contribute to some peak phage density that may or may not be sufficient to substantially decrease densities of target bacteria [93, 117]. Particularly, peak densities must exceed some minimum effective concentration to effect net reductions in bacterial densities and these densities can be achieved through a combination of supplying sufficient phage numbers per individual dose, supplying multiple doses, and/or allowing for phages to replicate* in situ*. Ideally phage densities will not be so high that toxicity results. Exactly what phage densities are necessary to achieve toxicities is not well appreciated, except that impurities in phage formulations can contribute to such toxicities (as too can potentially the humoral immune system given nonnaive patients). As a consequence of this uncertainty, what constitutes a preferred upper limit of phage densities is not known in the same way that minimum toxic concentrations can be appreciated for specific small-molecule drugs, except that this upper limit may be high relative to minimum effective phage densities. Lastly, various mechanisms exist whereby phage densities may decrease over time, which include what pharmacologically are described as metabolism and excretion, though as noted dosage dilution plays a role as well. A modified version of this figure is found in Abedon [93] as well.

These various pharmacological concepts require some modification to be fully applicable to phage therapy. First, movement into the blood is required only given systemic application and consequently often is not a goal with phage therapy, particularly of local infections. Second, movement for phages represents penetration to target bacteria and an important aspect of such penetration is into bacterial biofilms [85]. Third, “metabolism” for phages logically includes not just inactivation but also activation—particularly of phage bactericidal activity—and also the often-associated* in situ* amplification of phage numbers, where the latter can be described as an “auto dosing.” Auto dosing is not unique to phages but may be particularly effective for phages as antibacterial agents given that this amplification takes place in the immediate vicinity of target bacteria. Lastly and as is true for antimicrobial agents in general, the concept of “body” in pharmacology includes not only host tissues but also microorganisms, including target bacteria for antibacterial treatment. I provide elsewhere extensive review of these various concepts of phage therapy pharmacology [85, 91–93]. See Figure 4 for summary of a number of these pharmacological concepts as applied to phage therapy. See also Ryan et al. [94] and Parracho et al. [95] for additional consideration of pharmacology within the context of phage therapy and also M. E. Levison and J. H. Levison [90] for more general consideration of the pharmacodynamics and pharmacokinetics of antibacterials.

## 5. Phage Ecology

**Figure 5:**
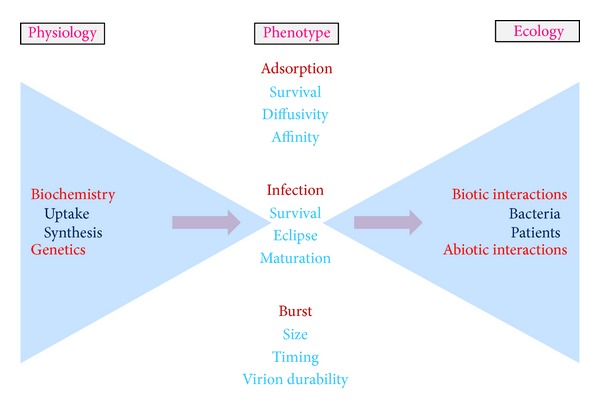
Molecular aspects of biology (left) give rise to organismal characteristics (middle) which in turn can give rise to ecological consequences (right). Ecological consequences include impact on environments as well as environment impact back onto organisms (not shown). Within phage therapy as a pharmacological process, these ecological consequences—with a patient's body serving as environment—can be viewed as being equivalent to considerations of pharmacodynamics (drug impact on body) and pharmacokinetics (body impact on drug), respectively. Physiology in turn is a description of how an organism's molecular aspects as well interactions with environments combine to give rise to organism functioning. Here physiological aspects are indicated, in the middle of the figure, particularly in terms of phage organismal properties. Phage physiology, within a phage therapy context, thus can be viewed as a highly complex elaboration on how chemical form, that is, of phages, gives rise to ecological properties (in terms principally of bacterial eradication), just as a pharmaceutical's chemistry gives rise to its pharmacological characteristics. Despite the complexity of a phage's chemical form as well as the process of translation of that form into so-called pharmacologically emergent properties, such properties as side effects can be less likely than the case with less-complex, small-molecule drugs. This often low phage propensity towards toxicity presumably is a consequence of phages consisting primarily of DNA (or RNA) and proteins that have been molded by evolution to be highly specific in their impact towards modification of bacterial metabolism and structure rather than that of eukaryotic organisms such as ourselves [66]. Note that this figure is modified from one found in Abedon [109].

Just as drugs can both impact and be impacted by bodies (pharmacodynamics and pharmacokinetics, resp.), organisms ecologically can both impact environments and be impacted by environments. Environments within the context of phage therapy include abiotic components, which are chemical or physical aspects especially of the extracellular environment, and also biotic components. The latter include target bacteria, nontarget bacteria, and also nonbacterial microorganisms, including other phages. The biotic environment in addition consists of the tissues associated with the patient being treated. Note that ecological interactions inevitably have underlying physiological bases, some of which for phages are as illustrated in Figure 5.

Within this context of biotic and abiotic components of environments, we can consider phage ecology from numerous perspectives including what can be described as phage organismal ecology, population ecology, community ecology, and ecosystem ecology [96–100]. These are the study of phage adaptations (a.k.a., evolutionary ecology), the study of phage populations such as in terms of their growth, the study especially of the phage impact on bacteria and* vice versa* (an aspect of community ecology), and the study of the phage impact on nutrient cycling, respectively. The latter particularly is a consequence of phage solubilization, via lysis, of nutrients that otherwise are associated with intact bacteria [101]. All of these are pertinent to considerations of phage therapy. In particular, and respectively, are the relevance of phage organismal properties to phage choice, the importance of phage population growth particularly when that is required for phages to collectively overwhelm and thereby subdue target populations of bacteria, the impact of phages on bacterial population dynamics, and solubilization in the course of phage-induced bacterial lysis of otherwise bacteria-associated toxins. The latter, among Gram-negative bacteria, includes most notoriously endotoxin [102–105].

**Figure 6:**
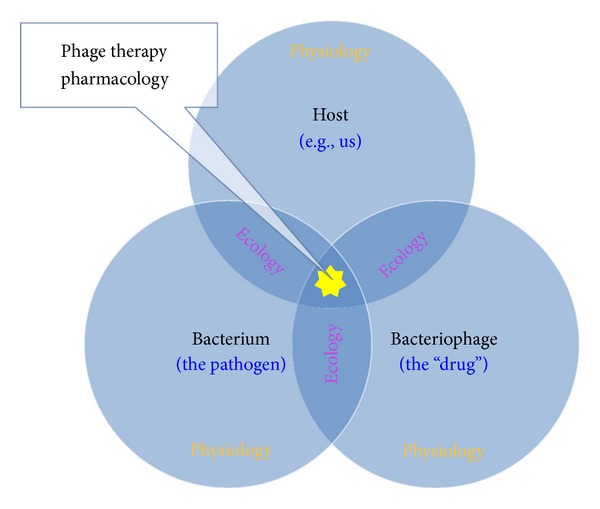
The interface between different organisms, and their physiologies, basically is ecological. The quantity of these interactions as well as their impact on physiologies increases with the number of organism types involved. With phage therapy, this includes three distinct species. (1) the patient, host, subject, or body that is experiencing a bacterial infection; (2) the infecting bacterial pathogen; and (3) the bacterial virus, a.k.a., phage or bacteriophage that is being used to treat the bacterial infection. Phage therapy pharmacology lies at the interface of these three components and thus inherently straddles both ecological and physiological considerations (with that confluence indicated by the star but not limited to the star). Since physiologies change with varying infection conditions as well as treatment approaches, including in terms of physiological adaptation to these changing conditions, phage therapy pharmacology can be viewed as being inherently eco-physiological.

Additional aspects of phage ecology include phage distribution, diversity, and numbers within environments, including environments consisting of phage-treated bodies. Important also is phage virion interaction with environments that are external to the individual being treated [95, 106]. See Figure 6 for facile illustration of how particularly it is ecological interactions, as mediated through the physiologies associated with multiple organisms that give rise to the pharmacology associated with phage therapy. See also Letarov et al. [107] for a complementary treatment of eco-physiological issues as they pertain to phage therapy. For general overviews of various aspects of phage ecology as well as phage impact on bacteria, see Abedon [108, 109].

## 6. Phage Virion Eco-Physiology

**Figure 7:**
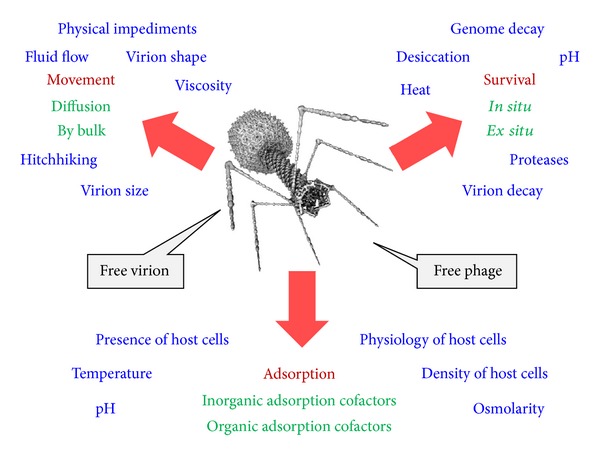
Phenomena impacting virions include their movement, their survival, and their adsorption. Shown also are various aspects of environments as relating to virion properties and functions (green text with subcategories in blue). The terms “free virion” and “free phage” are being used interchangeably in the figure.

Virions are not metabolically active. It does not follow, however, that they also are chemically inert. In fact, virions at a minimum play relatively active roles in the acquisition of cells, roles involving both docking (attachment) to cell surfaces and translocation of the virus genome past the cell's plasma membrane. As these are virion functions, they are aspects of virion and therefore of virus or phage physiology. As they also involve virion association with host cells, however, they at least arguably are aspects of the infection process itself. In this section, I concentrate instead on phage virion properties that exist when phages are not found in direct association with target bacteria. These include virion movement, bacteria-independent aspects of virion ability to adsorb, and virion resistance to inactivation also as seen in the absence of bacterial encounter. See Figure 7 for a summary of these various virion processes.

*Virion Movement, Penetration, and Titers*. To infect a cell, a virion must first encounter, that is, collide with a cell. The likelihood of such an encounter is impacted by a number of factors. These include the size of the bacterial target, the size of the virion, and the viscosity of the medium [110]. Additional relevant issues include whether various forms of nondiffusive movement are possible (i.e., fluid flow, bulk displacement of environments from one location to another, hitchhiking on animals, the phage administration process during phage therapy, etc.) and also whether any physical blocks on virion movement are present, including, for example, anatomical divisions between compartments as found in animals. These latter distribution issues are complex, varying as a function of phage properties, the site being treated, how phages are applied, and the extent to which disease as well as medical intervention may modify phage movement within bodies; see, for example, [85, 93, 107, 111, 112].

It is important, at a minimum, to consider during phage therapy the likelihood that a given target bacterium may be encountered by a phage. At its simplest, this likelihood can be described by a model that physicists describe as mass action [113]. While virion diffusion rates and bacterium target size are particularly important to resulting adsorption kinetics, what is most readily manipulated in the laboratory or clinic is phage density. Phage density is usually expressed as titers, often in units of plaque forming units present per mL [114, 115]. For phages, these titers, as found* in situ*, can vary as a function of how many phages are applied during phage therapy (dosage), how many phages reach target bacteria (a pharmacokinetic issue), and the potential for phages to replicate once they have reached target bacteria (also a pharmacokinetic issue).

Ultimately, the more phages that are present within the vicinity of a target bacterium, the greater the number of phage collisions that will occur with that bacterium per unit time. This impact of phage density on rates of phage-virion encounter in particular varies linearly with phage density. Phage densities, on the other hand, can vary over multiple orders of magnitude depending on the specifics of phage therapy treatment protocols. The rates at which bacteria are encountered by phages therefore can also vary by orders of magnitude, ranging from substantial as measured over seconds given high phage doses (e.g., 10^8^ phages/mL or more) to days or weeks or even longer at lower phage doses (e.g., 10^5^ phages/mL or lower) [45, 116]. Fortunately, phage* in situ* replication can offset failures to supply sufficient phage densities through traditional dosing, ideally supporting phage population growth to relatively effective densities such as in the range of 10^8^/ mL [93]. For further discussion of issues of phage adsorption during phage therapy, see Abedon [85, 92, 116, 117] as well as Abedon and Thomas-Abedon [91]. 

*Virion Adsorption Competency*. For infection, bacterial killing, and* in situ* phage amplification to occur, phage encounter with bacteria at a minimum must be followed by phage adsorption [113, 118–120]. To a first approximation, such adsorption is dependent upon complementarity between the phage adsorption proteins and the phage receptor molecules associated with the bacterial cell envelope [121]. If we assume that phages have been adequately matched to bacteria* in vitro*, prior to the initiation of treatment, then three eco-physiological, virion-adsorption-related issues nonetheless remain: whether the environment* in situ* can support phage adsorption, whether bacterial physiology* in situ* is such that receptor proteins for phage adsorption are expressed by target bacteria, and whether phage receptor proteins are present in sufficient numbers per bacterium to allow for reasonable rates or likelihoods of transition from phage encounter with a bacterium to phage adsorption [56]. An additional issue is that even when phage growth is effective* in vivo*, that does not necessarily always translate into antibacterial efficacy [122].

Generally phage adsorption is dependent on environmental pH, temperature, osmolarity, adsorption inhibitors, and also the presence of what are described as phage adsorption cofactors [56, 123]. The latter often include specific cations, whether monovalent (e.g., sodium ions) or divalent (e.g., magnesium ions), though also can include organic cofactors. Particularly well studied among organic adsorption cofactors, at least historically, is free tryptophan's role in T-even phage adsorption [124]. In the absence of adsorption cofactors, virions are unable to correctly interact with bacterial cells. For consideration of these issues within the context of phage-mediated biocontrol or phage therapy, see [125, 126]. 

*Virion Survival.* There traditionally are two basic mechanisms that lead to reductions in a drug's concentration* in vivo*. These are metabolism and elimination. Metabolism is the chemical modification of drugs, typically though not always towards reduction in drug activity (e.g., as mediated by the liver). With phages, such inactivation is seen in terms of the immune system's impact, which includes both innate actions and the actions of antibodies, but inactivation also can occur in the course of infection of otherwise resistant bacteria. Elimination as a means of phage depletion from the body, particularly by the kidneys, by contrast is not considered to play as large role in phage loss [107], though this route has been explored by a number of authors [111, 127–131].

**Figure 8:**
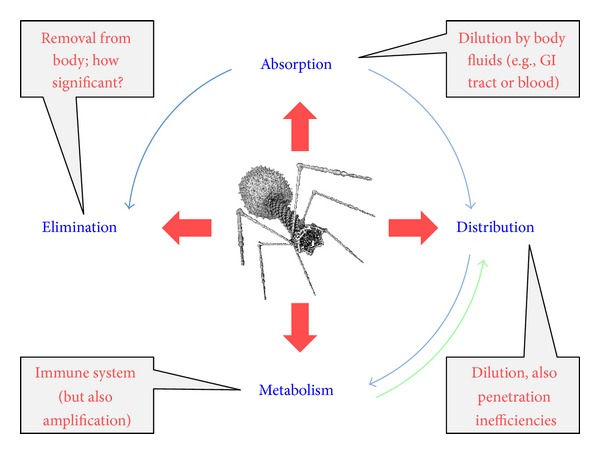
Impacts of pharmacokinetic phenomena during phage therapy. These processes occur in approximate temporal order as indicated by blue or inner arrows. Metabolism, via amplification of virion numbers, is also shown as contributing to increases in phage numbers that then may be distributed to other locations or compartments within the body (green or outer arrow). Amplification can also give rise to increased phage titers in blood, though this is not indicated. Also not indicated is activation of phage bactericidal activity, which can be viewed as an aspect of metabolism and one that precedes amplification (though which leads to amplification only given successful lytic, productive infection). Note that absorption and distribution too have the effect of increasing phage concentrations in specific compartments, the blood and nonblood tissues, respectively, as these are a means by which access to these compartments is achieved. These increases in concentration, however, are not relative to the initial phage dose but instead are relative to concentrations within compartments as observed prior to dosing.

Less obvious means of reduction in virion concentrations, as considered in a traditional pharmacokinetic manner, are absorption and distribution. Particularly, simply the application of phages, if done systemically (i.e., as resulting in absorption) as well as orally to the gastrointestinal tract has the effect of diluting those phages and thereby reducing phage concentrations relative to those found in the original dose. Distribution of phages to nonblood body tissues too has the effect of diluting virions and therefore reducing concentrations at least relative to those concentrations found in the blood, though it also has the effect of increasing concentrations in receiving tissues such as to densities that are greater than zero. The end result is that phage concentrations at sites of application typically will be greater, and potentially substantially so, than they will be at their site of action. This can be less of a concern the closer that sites of application and action are associated and particularly so if the two are identical—site of administration and site of action—as often is the case given local treatment such as of wounds. Alternatively, it is especially* in situ* phage amplification, as can occur in association with phage infection along with subsequent bacterial killing, that can counteract depletion in phage densities. Not to be overlooked, the traditional approach in pharmacology to addressing issues of drug* in situ *depletion is to provide additional drug, or phages [93, 117], in multiple doses over the course of treatment. See Figure 8 for summary of especially the impact of these various pharmacokinetic processes during phage therapy.

Phage virion interaction with and inactivation by antibodies has been long studied [86] and is a potential concern particularly given repeated or long-term phage systemic application [132]. Relevant to whether such activity is even an issue for phage therapy, however, are the following points. (i) In practice, antibody-mediated inactivation of phage virions during phage therapy does not appear to be recognized as an important issue [107], (ii) phages are diverse in terms of their immunogenicity such that application of one phage isolate to an animal will not necessarily result in the production of cross-reacting antibodies to another phage isolate [133], (iii) systemic application of phage types to which a body can mount a severe immune reaction is problematic for reasons that go well beyond the issue of virion survival though with phages such severe immune reactions following systemic application nonetheless do not necessarily occur [134], (iv) organisms actually are exposed over their lifespans to numerous phages both topically and systemically and there is no evidence that this has a significant impact on health [87, 107], (v) application of phages* per os* (orally but with systemic intentions) seems to reduce the immunogenicity of phage formulations [135], (vi) for tailed phages, only a small fraction of virion surfaces represent epitopes through which neutralization can be effected such that not all antibody reactions will result in phage inactivation [107], (vii) virion inactivation is less of a concern given local rather than systemic phage application, and (viii) one approach to combatting phage losses is to simply supply more phages [136].

Phage losses also can occur via the action of what is known as the mononuclear phagocyte system, previously described as the reticuloendothelial system [137, 138]. The result of this clearance can be less than immediate inactivation of phage virions, resulting instead in virion accumulation such as in the spleen. This does have the effect of sequestering phages away from target bacteria, however, which in effect is similar to phage inactivation. Removal of virions by the mononuclear phagocyte system is mediated by certain protein determinants associated with virions, and phage populations can be enriched for variants possessing less immunogenic determinants. This selection is accomplished via injection into animals and then later plating for phages remaining in the blood. The mechanism of removal by the mononuclear phagocyte system is not mediated by antibodies, is relevant particularly to phage systemic application, does not occur instantaneously so is not a complete block on phage distribution following access to systemic circulation, and to a degree can be countered by supplying more phages. Though as noted phage mutations that allow bypassing of the mononuclear phagocyte system do exist, it is not certain the extent to which such mutations are effective in different species from which they were generated (e.g., mice versus humans) nor whether they are consistently effective across genetically diverse individuals found within the same species. See also the discussion by Goodridge [139]. An additional though potentially unrelated issue is that phage treatment has been observed also to generate positive immunomodulatory effects [135].

## 7. Virion-Body Interactions

**Figure 9:**
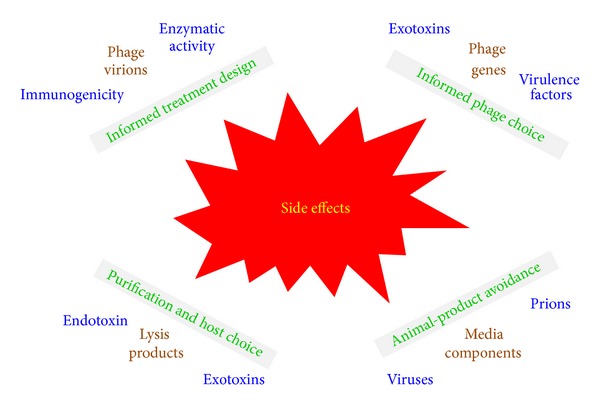
Summary of secondary pharmacodynamic concerns that are peculiar to phage- or protein-based antibacterial therapy. Issues are listed in brown at the corners of the figure, with more specific considerations listed in blue. Means by which these issues can be mitigated are presented as green blocks of text as found between concerns and the indication of  “Side effects” shown in the center. Thus, for example, the issue of bacterial lysis products in phage formulations may be mitigated through a combination of informed bacterial choice in some combination with sufficient post-lysis purification of resulting phages.

The interactions between virions and patient bodies fall into two basic categories, corresponding explicitly to pharmacokinetics versus pharmacodynamics. That is, the impact that bodies have on phages (pharmacokinetics), particularly on the densities of functional as well as available virions, versus the impact that virions can have on bodies (pharmacodynamics). As issues of phage infection of bacteria are considered in a subsequent section and the pharmacokinetics of virion losses were outlined above, here it is secondary pharmacodynamics that are briefly considered. Specifically we consider evidence of phage-formulation toxicity to patients. This can be toxicity effected by virions themselves, anaphylactic immune reactions to virions, and toxicity of nonphage carrier material. The latter particularly would be the presence of lysis products that have not been removed in the course of phage-virion purification. See Figure 9 for summary.

Though such side effects can be associated with any protein-based drug, there nonetheless is little evidence of any inherent toxicity associated with phage virions. By contrast, there unquestionably can be toxicity associated with the bacterial lysis products that are produced in the course of phage-product manufacturing, potentially including bacterial exotoxins if those are generated by the bacteria used to produce phages. Also of concern is endotoxin that is solubilized upon lysis of Gram-negative bacteria [136, 140–142]. Generation of these lysis products* in vivo*, during treatment, is an issue associated with any lytic antibacterial and certainly should be taken into account during the design of phage therapy protocols and/or avoided via the use of lysis defective phages [139, 143].

In terms of phage formulations themselves, it is best to avoid ingredients such as animal-based media components that also could carry toxic or infectious materials, to avoid producing phages using hosts that produce exotoxins, and also to appropriately purify virions once produced [142, 144, 145]. Use of phage products that are less fully purified, however, can be permissible for topical rather than systemic application. See Miedzybrodzki et al. [64] for recent discussion of potential side effects—as associated with impurities in formulations, immune reactions, or both—that have been shown to occasionally arise in the course of phage therapy treatment; see too the discussions by Parracho et al. [95] and by Henien [83]. See Merril et al. [134] along with other publications by this same group for broader discussion of the phage potential to interact with animal tissues.

## 8. Phage-Bacterial Interactions

In pharmacology, the concept of “body” includes not only body tissues but also an organism's microbiota, but intentionally left out of that description is the word, “normal.” This is because pharmacology obviously addresses drug impact on disease. Targeted bacteria during phage therapy thus qualify as “body” components as do also nontarget bacteria. Ecologically, bacteria are considered to be components of communities of organisms, that is, consisting of multiple species found in approximately the same location, and phage-bacterial interactions also can be considered under the heading of what is known as community ecology. Physiologically, most of phage metabolism takes place within the explicit context of phage-bacterial interactions. In considering the eco-physiology of phage therapy pharmacology, these phage-bacterial interactions thus hold a prominent position. In this section, I consider these particularly in pharmacological terms.

*Adsorption and Affinity.* The concept of phage adsorption combines consideration of virion diffusion, bacterium encounter, virion attachment to bacteria, and also, depending on author, phage genome translocation into the bacterial cytoplasm. A primary component of that adsorption interaction is virion affinity for the surface of target bacteria, which to at least a first approximation is equivalent to the affinity that drugs have for their targets as well as the sensitivity that bacteria have to specific antibacterials. Such affinity and/or sensitivity is a component of an antibiotic's minimum inhibitory concentration (MIC).

Though MIC is not as easily defined for phages as for antibiotics, affinity is a component of a phage's adsorption rate constant, that is, the per-unit-time likelihood of a phage's adsorption to a bacterium. The adsorption rate constant helps determine a phage's minimum* effective* concentration as an antibacterial agent as well as other measures of a phage's virion-density-dependent impact on target bacteria [117]. See, though, a recent study addressing this issue of MIC determination for bacteriophages [146] using what can be described also as an* in vitro* phage virulence test [41]. Phage affinity for bacteria—ranging from zero affinity to a likelihood of adsorption of unity given phage collision with a bacterium—therefore represents a key and to a large degree defining characteristic of phage's potential to be used therapeutically against a given bacterial strain.

*Phage-Infection Productivity*. For phage therapy, phage infections must be bactericidal and ideally productive as well, that is, producing and then releasing new phage virions. Poor bacterial growth conditions and/or bacteria that have reached an approximation of stationary phase, however, can reduce phage infection productivity, though the impact varies from phage to phage [2, 147, 148]. Little is understood, though, about how differences in bacterial physiology* in situ*, such as during phage therapy, may affect phage productivity relative to that seen* in vitro* [134, 136]. Speculation is possible, however.

Very dense bacterial cultures and/or bacteria that have reached stationary phase as may be found in biofilms [85, 149], for example, may be less able to support highly productive phage infections, and potentially may be refractory even to phage-mediated bacterial killing [150]. Perhaps consistently, debridement can be beneficial towards apparently active phage treatment (see below) of wounds [151], perhaps by improving bacterial physiology within infected tissue such that bactericidal as well as productive phage infections are more readily supported. On the other hand, log-phase bacterial pathogens growing in association with human tissues may very well have access to sufficient nutrients to display physiological states that are comparable to that readily observed in rich media* in vitro*.

These basic considerations of the impact of bacterial physiology on phage performance may date back to d'Hérelle. From his 1930 book (as translated to English at the time by Smith) [152], d'Hérelle argued that phages can be much more effective against acute bacterial infections versus more chronic infections. Indeed, he suggests (p. 170, italics his) that “in acute diseases, it is sufficient to apply,* as soon as the first symptoms are noticed,* or early during the course of the disease, a small quantity of a potent “stock” bacteriophage in order to occasion the destruction of the bacteria and thus bring about recovery.” Under these conditions, bacterial physiology presumably is closer to that of log phase and/or bacterial biofilms have not yet become fully established, resulting, given proper phage choice, in more productive phage infections and/or more bacterial killing. With chronic infections by bacterial pathogens, by contrast, he argues (p. 176) that “it may be necessary to continue bacteriophage therapy over a relatively long period.” That is, under what may present as more stationary phase bacteria and/or bacteria that have more fully established biofilms, it tends to be necessary to supply phages in multiple doses over much longer periods, for example, weeks [43, 153, 154] versus days for less established infections.

The issue of the degree to which a given bacterium can support phage productivity is particularly relevant if phage population growth is required to support phage treatment [122, 126, 155]. If that is not the case, then simply phage-mediated bacterial killing, perhaps in combination with phage-mediated bacterial lysis, may be sufficient to achieve phage therapy success. These issues are more than academic as they go to the heart of a number of pharmacologically relevant questions in phage therapy [93, 117]. How many phages should be applied per dose? How many doses should be applied in the course of treatment? And how often should doses be applied? In particular, the lower the potential for* in situ* bacteria to support large numbers of robustly productive phage infections, then the more phages that may need to be applied, per dose or over multiple doses, to effect meaningful reductions in bacterial counts. For circumstances where reaching target bacteria with higher phage densities is impractical, however, treatment success can be dependent instead on the productivity of resulting infections.

Standard means of assessment of phage host range, particularly by what is known as spot testing [114], can fail to recognize poor infection productivity. Plaque formation, alternatively, does provide evidence of at least minimal levels of infection productivity [156], though plaque formation can vary in likelihood as a function of plaquing conditions [59, 157, 158]. Direct determination of phage burst size [119], by contrast, does measure phage-infection productivity, though none of these methods explicitly describe phage infection productivity as it may appear* in situ*. Assumptions of phage productivity, bactericidal activity, or even biofilm disruption as occurs* in situ* relative to* in vitro* thus might be questioned should seemingly adequate phage dosing nonetheless result in treatment failure.

*Metabolism (Pharmacokinetics).* Body impact on a drug's chemical structure, as a pharmacokinetic process, is described as metabolism. Metabolism for many drugs represents inactivation as mediated, for example, by liver enzymes. For a few drugs, however, these chemical changes result instead in increases in activity [159]. The metabolic impact of bacteria on drugs also can include drug inactivation, particularly as associated with bacterial resistance [160]. This bacterium-mediated inactivation is as one sees, for example, via the action of antipenicillin *β*-lactamase enzymes [161].

Immune responses, as considered above, can have an equivalent impact on phage virions, though in the short term, antivirion immunity does not necessarily correspond to actual virion chemical modification so much as a physical blocking or sequestration of activity. Phage adsorption and subsequent infection of bacteria, alternatively, do inherently give rise to phage chemical modification, though the result of this modification can vary depending upon phage, bacterium, and circumstances. Specifically, phage adsorption to a bacterium, from the perspective of metabolism as a pharmacokinetic phenomenon, can consist of inactivation or activation, both particularly in terms of a phage's cytotoxic activity, and also subsequent amplification of phage numbers. Metabolism as displayed by a patient's actual body tissues, by contrast, tends to result solely in phage inactivation rather than activation. Specifically, phage cytotoxic activity requires activation, and that activation is achieved solely in the course of infection of specific bacterial types, and that cytotoxicity also predominantly targets those bacteria being infected. Phage therapy as a consequence can display an inherently lower toxicity than can be achieved by many small-molecule antibacterial agents, including many antibiotics [162, 163]. Small-molecule agents, that is, often can more readily interact with non-target bacteria or body tissues in a physiologically active form than can phage particles.

Alternatively, when infecting phages are sensitive to a bacterium's abortive infection system, both phage and bacterium do not survive. In terms of metabolism, the bacteriophage nonetheless has been activated as an antibacterial agent in the course of these interactions since, upon infection, it has become able to display bactericidal activity [17, 164]. Such a phage is acting equivalently to a small-molecule antibiotic, though one that becomes activated only upon adsorption to a target bacterium. Productive infections by lytic phages, by contrast, involve not only phage activation as a cytotoxic antibacterial agent but also chemical modification such that amplification in phage number occurs.

*Additional Abortive Infection-Like Mechanisms*. Results that are equivalent to the impact of bacterial abortive infection systems on phages can be engineered into either phages or phage therapy protocols. One means is through the genetic engineering of phages so that they are bactericidal but not productive [139]. Alternatively, it is possible to treat phages, such as with ultraviolet radiation, so that again they are bactericidal but not, at least to a degree, capable of producing phage progeny [165]. In addition, phage-like but not replicative bacteriocins may be employed, such as R-type pyocins which are active against* Pseudomonas* [166, 167] but which also may be engineered to recognize, for example,* E. coli* O157:H7 [168, 169]. Replication incompetent phages in principle should not be capable of transducing bacterial genes between target bacteria, such as genes encoding bacterial virulence factors [165, 167]. If combined with blocks on phage-induced bacterial lysis, then these efforts also can have the effect of targeting and then killing bacteria but without releasing toxic lysis products.

In terms of modification of phage therapy protocols rather than of phages themselves, a process known as lysis from without [170] can give rise to similarly abortive results, with phages displaying bactericidal activity without subsequent phage replication. The degree of phage chemical modification required for phages to effect lysis from without, however, can be less than that of an abortive infection since actual phage infection is not required for lysis from without, just adsorption. The process specifically is effected by supplying to target bacteria extremely high phage densities, for example, such that on the order of 100 virions adsorb each targeted bacterium. This process, though, has the effect only of blocking phage amplification rather than also blocking bacterial lysis and can otherwise be somewhat redundant in terms of antibacterial activity since a lytic infection initiated by a single adsorbing phage typically is sufficient to effect phage bactericidal activity. In addition, only a subset of lytic phage types in fact may be physiologically able to effect lysis from without and little effort has been extended to distinguish among phages in terms of this ability.

Such efforts—blocks on phage replication, blocks on phage-induced bacterial lysis, reliance solely on lysis from without to effect bacterial killing, or use of bacteriocins rather than phages—all have the effect either of reducing or eliminating auto dosing. This can increase control over dosing by at least conceptually simplifying antibacterial pharmacokinetics but does so at the expense of “drug” amplification* in situ* at the site of infection. Nonetheless, it is important to keep in mind that antibacterial drugs generally function in this same manner; that is, either sequestration or loss of drug molecules occurs in the course of their display of antibacterial activity, and this occurs without subsequent “auto” amplification of drug activity. Exceptional among antibacterial agents therefore are bactericidal but nonabortive phage infections since their* in situ* infection of target bacteria gives rise to new phage particles in the course of their effecting antibacterial activity.

*Passive versus Active Treatment.* A drug's impact on either body tissues or associated microbiota can be as intended (primary pharmacodynamics) or in some manner unintended, particularly as side effects (secondary pharmacodynamics). For phage therapy, primary pharmacodynamics are associated predominantly with the negative impact of phage infections on target bacteria, particularly activation of phage bactericidal activity (see previous section). Not all phage infections are equivalent with regard to their negative impact on bacteria, however, and therefore are not equivalent in terms of their primary pharmacodynamics. Key issues include the number of phages that must be supplied per dose to achieve a given treatment outcome, the frequency of dosing that is necessary, rates with which phage virions acquire bacteria (such as described by the phage adsorption rate constant), the likelihood that phage adsorption will result in bacterial elimination, whether or not phages induce bacterial lysis, and the contribution of infections, especially of target bacteria, to an amplification of phage numbers. The latter, ecologically, can be described simply as* in situ* phage population growth.

The importance of these various issues can differ as a function of the specifics of treatment protocols. Of particular importance is how many phage virions can be delivered to the vicinity of target bacteria via traditional approaches to dosing, and to what extent bacterial “elimination” is required to achieve infection clearance. In general, the more phages that can be delivered to the site of an infection then the less relevant adsorption rate constants or rates of* in situ* phage population growth can be to treatment success. In addition, the more accessible that bacteria are to phage adsorption then the less important that bacterial lysis—or otherwise modification of the bacteria-containing tissues such as via debridement [20]—may be to achieve infection clearance. Specifically, if phages do not need to lyse bacteria to penetrate to additional bacteria, then lysis can be less important to bacterial eradication than if such lysis instead does play a role in phage penetration to target bacteria. Similarly, if hydrolysis of extracellular polymeric substances associated with biofilms is not required for phage-mediated clearance of those biofilms, then such hydrolysis will be less crucial to treatment success [85, 171–176].

It is possible to frame these issues in terms of what can be described as passive treatment, active treatment, and/or active penetration [91]. With passive treatment, also known as inundation therapy, sufficient densities of virions are supplied via traditional approaches to dosing to result in phage adsorption of a majority of target bacteria, ideally a vast majority. It then is subsequent phage-mediated bacterial killing, followed by immune-system removal of resulting debris and/or of still-viable bacteria [49], that clears the bacterial pathogen and associated infection. Such inundative quantities of phages in principle can be supplied via only a single dose but legitimately may be supplied instead over multiple doses. Importantly, the rate of phage acquisition of bacteria will be determined in the case of passive treatment by a combination of the phage adsorption rate constant, the number of phages supplied, and the potential for supplied phages to penetrate to target bacteria, with the latter such as into biofilms but also for systemic treatments in terms of phage absorption as well as distribution about the body more generally.

Passive treatment is absolutely essential for phage therapy success if bacteria for whatever reason are unable to support phage population growth to inundative densities, that is, to support active treatment. Failures to support adequate phage population growth may be due to excessively low densities of target bacteria and/or because of conflicts between bacterial* in situ* physiology and phage replication upon bacterial infection. Passive treatment does require that phage activation as an antibacterial agent efficiently occurs upon phage adsorption to target bacteria. At the same time, however, it does not necessarily imply absence of productive phage infection and subsequent phage population growth. Passive treatment instead is defined simply as being dependent on phage antibacterial activity but* not* on phage* in situ* amplification. Passive treatment in addition, and as noted, also need not result in the lysis of target bacteria, though such lysis may be required for phages to effect clearance of biofilms (re: active penetration).

**Figure 10:**
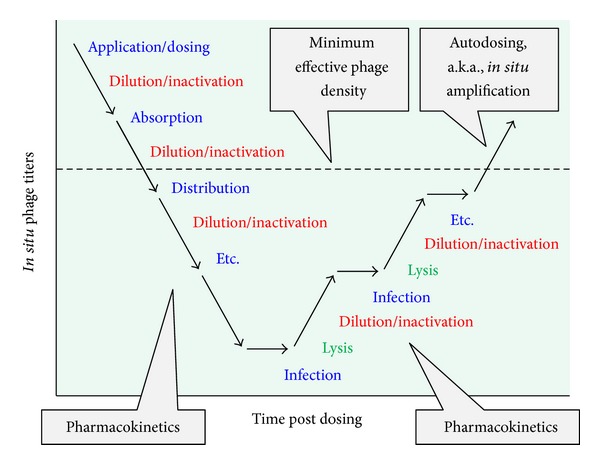
Time course of the pharmacokinetics of active phage therapy. Numerous pharmacokinetic processes–either through dilution, inactivation, or inefficiencies in penetration–have the effect of reducing phage densities* in situ* such as below minimum effective phage densities (MEPDs). These losses may be minimized by reducing the length of the chain of processes separating phage application from phage contact with target bacteria or instead can be addressed by supplying more phages, such as to counteract inevitable losses. Metabolism as a pharmacokinetic process, in the form of phage replication and therefore* in situ* amplification in density (auto dosing), can reverse these losses and allow an achievement of MEPDs, at least local to target bacteria. The process illustrated in the figure is an elaboration on the concept that otherwise has been described as active treatment, that is, supplying insufficient phage numbers through traditional dosing to achieve MEPDs, and thus relying on* active* phage replication instead to achieve these densities.

With active treatment, these latter issues, particularly in terms of phage population growth, are by contrast crucial to treatment success. That is, active treatment, essentially by definition, is phage therapy that is dependent on* in situ* phage population growth to achieve sufficient phage titers to effect adequate levels of bacterial killing (Figure 10). Active treatment in addition can be associated not only with active phage population growth,* in situ*, but also with active phage penetration into bacterial biofilms. Phages in this case not only are lysing target bacteria but also are supplying additional phages that can effect further penetration into biofilms. This scenario contrasts that of purely passive treatment, where bacterial lysis likely is still required to achieve active penetration into biofilms but in principle additional phage quantities can be supplied from exogenous rather than endogenous sources. Further complicating this issue, note that exogenously supplied phages also can be provided repeatedly over the course of active treatment; phage* in situ* population growth, that is, is not the only means by which phage densities may be sustained at relatively high levels over the entirety of a treatment protocol.

**Figure 11:**
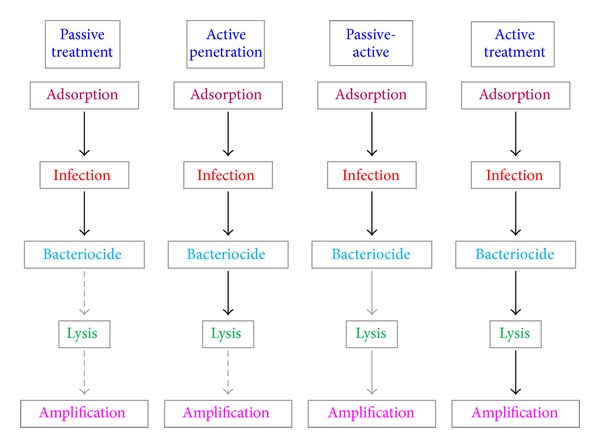
Comparing proximate outcomes during different categories of phage therapy progress. In passive treatment, only cell killing must occur as a proximate outcome, though cell lysis as well as* in situ* amplification of phage numbers may occur as well. By definition, though, they do not have to happen for passive treatment to successfully clear a bacterial infection (hence use of dashed, grayed arrows towards the bottom of this column). Phage active penetration into bacterial biofilms appears to be dependent on some form of phage enzymatic activity other than those required to physiologically or genetically kill bacteria. Here this is indicated as bactericidal infection occurring in combination with bacterial lysis, with the latter contributing to further phage penetration into biofilms and/or improved phage-infection physiology. Such phage activity potentially may also improve antibiotic [223] or disinfectant [224] penetration into biofilms or at least their effectiveness against biofilms. Phage* in situ* amplification, though potentially helpful towards further phage penetration, nonetheless in this case is not necessarily absolutely required (dashed, gray arrow). Payne and Jansen [52] describe an intermediate state between active and passive treatment that they term “mixed passive/active” (here, for clarity, “passive-active”). This treatment approach involves a combination of dosing with large numbers of phages and subsequent phage population growth. It is the opinion of this author that this latter approach, possibly in combination with multiple dosing, likely either should or does represent a default approach to effecting phage therapy treatments. This represents supplying relatively large phage numbers to bacterial infections—in single or multiple doses—though nonetheless supplying phage numbers that are less than completely overwhelming (i.e., less than completely inundative) with the assumption that phage* in situ* population growth will enhance those numbers local to either planktonic bacteria or instead bacterial biofilms or microcolonies. See Abedon [225, 226] for consideration of the latter. The reduced but not eliminated requirement for lysis and amplification in the case of “passive-active” is indicated using solid but gray arrows rather than dashed gray arrows. Lastly, active treatment by definition is dependent on both lysis and* in situ* phage amplification (black, solid arrows).

These roles played by bacterial lysis along with* in situ* phage amplification are summarized in Figure 11. A conclusion is that it can be possible to “get away” with treating infections with fewer phages than are actually required to achieve bacterial eradication so long as sufficient phage population growth can occur* in situ*. Such population growth, however, may not be necessary for phage-mediated clearance of biofilms, though some kind of antibacterial activity that is in addition to simply killing bacteria may be useful. Such additional bacterial activity can include, for example, phage-induced bacterial lysis. Alternatively, when phage treatments fail to successfully clear bacterial infections, there are at least three general issues that should be considered as possible causes for this insufficient treatment success: less bactericidal activity* in situ* than may be required (and/or less structural decimation of bacterial biofilms), less effective phage penetration to target bacteria, or insufficient amplification of phage numbers following contract with target bacteria. It is possible that all three of these issues may be addressed at least in part by supplying more phages per dose during phage treatments, as well as more doses over time, thereby biasing the supplying of phages more towards that provided with purely passive treatment strategies. For additional discussion of how one might go about debugging phage therapy protocols, see Abedon [92].

*Spectrum of Activity.* The spectrum of activity of antibacterial agents is that range of microbial targets against which they are effective. This can include all bacteria, a large subset of bacteria (e.g., Gram-positive bacteria), a particular bacterial genus, species, or even a collection of related strains. One describes the more inclusive end of this spectrum as broader and the less inclusive end as narrower. Though economic incentives in earlier years for antibiotics may have biased development towards drugs possessing broader activity, that may be changing both as new broader spectrum antibacterial drugs with sufficient selectivity in their toxicity have become scarce [177] and also as the utility of limiting drug impact on normal flora bacteria becomes better recognized [178–180]. See Then and Sahl [181] for general discussion of the utility of antimicrobial agents possessing narrower rather than broader spectra.

There is also a difference between clinical sensitivity and sensitivity to a drug as observed in the laboratory. During antibiotic treatment, there usually will be limits to the concentrations of a drug that can be achieved at the site of intended activity. These limitations will be due either to delivery (pharmacokinetic) issues, side effects (which are secondary pharmacodynamic issues) [182], or physical, chemical, or other issues associated with formulations (e.g., drug precipitation could occur at too high concentrations, or simply be too costly to apply in large amounts). The result is that a drug's spectrum of activity, as actually used, typically will be narrower than what otherwise might be attainable in the laboratory. Alternatively, there often exists motivation to achieve relatively high rather than relatively low drug densities* in vivo*, during use, whether for the sake of assuring efficacy [183] or to increase the length of intervals between dosing. There often is tension in terms of what drug concentrations are achieved, in other words, between requirements for higher concentrations for drug efficacy (primary pharmacodynamics) and other drug properties which serve to limit what* in situ* concentrations are possible, and this tension can be seen in part in terms of an antibacterial drug's spectrum of activity.

**Figure 12:**
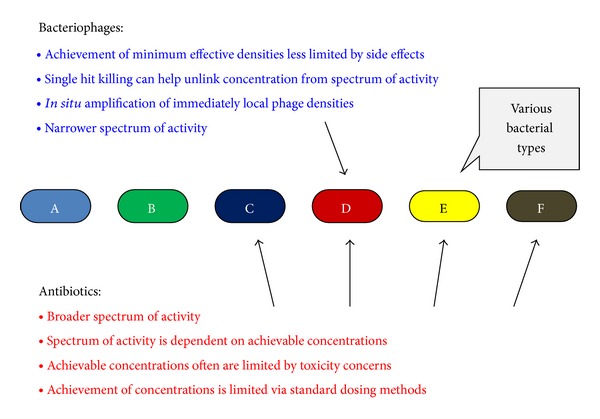
First-approximation comparison of bacteriophages and antibiotics in terms of their activity spectra in combination with various concentration and dosing issues. In short, phages tend to display narrower activity spectra but that activity can be less dependent on concentration issues, particularly given passive treatment, than the activity spectra displayed by small-molecule antibacterial agents.

The spectrum of activity of a phage as an antibacterial agent is its host range and phage host ranges tend to be much narrower than those of typical antibacterial drugs, often limited to approximately one bacterial species [17]. This property is beneficial to the extent that it is primarily targeted bacteria that will tend to be affected by applied phages, or at least only a relatively small subset of normal microbiota bacteria in the case of opportunistic pathogens that otherwise are present as commensal organisms. It also implies that the size and diversity of the population of bacteria being subjected to antibacterial-mediated natural selection are smaller given phage treatment versus treatment using more broadly active antibiotics, though whether that has an impact on resistance evolution or otherwise can impact the outcome of individual treatments is not certain. Narrowness of an antibacterial's spectrum of activity, though, can result in a requirement for greater care by physicians in selecting agents to use against a specific bacterial target than tends to be the case with broader spectrum antibacterials, such as is the case for many antibiotics [73, 74]. See Figure 12 for a first-approximation consideration of various properties of phages versus antibiotics that can be observed in the course of treatment of bacterial infections. In the section that follows, however, I consider how especially bacteriophage properties, as listed, can interact in ways that can be relevant from the perspective of phage spectrum of activity to the design of phage therapy treatment protocols.

*Phage Spectrum of Activity as a Function of Phage Concentration*. Will phage spectrum of activity, like that of antibiotics, also vary with density? There actually are at least three answers to that question. The first answer stems from phages generally displaying single-hit killing kinetics [184]. As a consequence, for specific target bacteria that have become adsorbed by at least one bactericidal phage, the overall phage concentration has little bearing on that bacterium's survival. By contrast, if a bacterium must encounter, say, 1000 antibacterial molecules for substantial antibacterial activity to occur, then sensitivity will vary in a saturable manner with antibacterial concentration. Bacteria, that is, can be partially inhibited by antibiotics but for phages inhibition is much more binary with, for the most part, bacteria either killed by phage adsorption or not.

Passive treatment, as noted, is dependent on phage adsorption to target bacteria, with that adsorption followed by activation of phage bactericidal activity. Though spectrum of activity in terms of bactericidal activity will not be expected to vary with phage concentration, rates of bacterial adsorption and therefore of bacterial killing will. The spectrum of activity of a given passive treatment protocol consequently could vary with phage concentration, with more adsorption-susceptible bacteria more likely affected by a given phage dose than less adsorption-susceptible bacteria. A simple means of countering this latter concern is simply to dose with greater phage numbers. Achievement of phage titers of 10^8^/mL or even more at the site of infections, for example, is not considered to be problematic in terms of the potential generation of side effects. Such concentrations, particularly as sustained locally over the course of treatments, typically should be adequate to achieve bacterial clearance. See Abedon [93] for defense of 10^8^ phages/mL as a reasonable target for local phage densities, achieved via either active or passive means, towards successful treatment outcome, which in turn is roughly consistent with arguments and evidence supplied by the following publications [45, 50, 85, 91, 116, 117, 119, 185–190]. Note, though, that for particularly poorly adsorbing phages the achievement of phage densities that are even higher than 10^8^/mL at their site of activity may be necessary to realize adequate bacterial killing. As a function of adsorption susceptibility, therefore, the spectrum of activity of phages indeed may vary with concentration, and particularly so as a function of a phage's ability to reach and then infect target bacteria* in situ*.

The third point concerning phage spectrum of activity stems from issues of active treatment. For active treatment to be effective, phages not only must adsorb target bacteria and then be activated to display bactericidal activity, but also, by definition, must increase their numbers to inundative densities in the course of infecting these same or related target bacteria. In terms of active treatment, phage spectrum of activity therefore will be defined by all three of these parameters. Active treatment thus can fail even given reasonable ability by a phage to adsorb and then kill target bacteria. Furthermore, contrasting passive treatment, there are unequivocal though mostly conceptual limits to what phage densities may be applied to infections in the course of active treatment. At an extreme, a phage that is unable to replicate while infecting a specific target bacterium will not succeed in eradicating an infection unless inundative phage densities are supplied by standard dosing means. Inundative phage densities supplied without auto dosing however represented a passive rather than active treatment strategy. Therefore, while the impact of densities on spectrum of activity may be less constraining for phages in comparison to antibiotics, reliance on active treatment, whether that reliance is intentional or instead by necessity, in fact may result in phage concentration-dependent limitations of phage spectrum of activity. Such limitations, though, may be addressed in many instances via the employment of mixtures of multiple phages possessing different activity spectra, that is, phage cocktails as therapeutic reagents [73, 74].

## 9. Phage Interaction with Already Phage-Infected Bacteria

**Figure 13:**
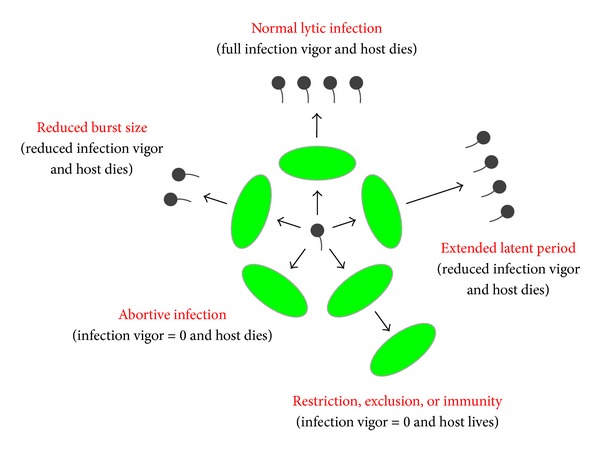
Ecological as well as physiological perspective on bacterial resistance to phages as seen following phage adsorption. Specifically, there exist gradations in bacterial interference with phage productivity ranging from (i) no interference (“normal lytic infection”) to (ii) partial blocks on phage productivity and/or extension of the phage infection cycle that can slow down phage population growth (“reduced infection vigor” [17] as seen with “reduced burst size” or “extended latent period”) to (iii) bacterial self-sacrifice for the sake of phage elimination (“abortive infection”; see also [225]) to (iv) bacteria simply inactivating infecting phages but without loss of bacterial viability (“restriction, exclusion, or immunity”; hosts in any case are shown as green ovals). These various mechanisms are reviewed by Hyman and Abedon [17] and also Labrie et al. [164]. Not shown, bacteria can also block phage infection by resisting phage attachment following phage encounter, though generally this does not result in phage metabolism in either a physiological or pharmacological sense. For comparison of mechanisms of bacterial resistance to phages to the immunity displayed especially by animals against pathogens, see Abedon [227]. Note also that analogies exist between mechanisms of bacterial resistance to phages and mechanisms of bacterial resistance to antibiotics. These include as mediated by compound destruction or avoidance of interaction through changes in target structures, though notably absent is a phage-resistance equivalent to “efflux off the antibiotic from the cell” (p. 1451) [160].

In addition to interacting with target bacteria and to a more limited extent other organisms such as ourselves and our non-target microbiota, phages also can interact with other phages. This can include interactions between phages that are closely related or with ones that are less so. Interactions occur predominantly following phage adsorption to already phage-infected bacteria and the* primary* phage infection may effect defense mechanisms against the* secondary* phage, such as superinfection exclusion (SE) or superinfection immunity (SI). These are the blocking of phage entrance into the bacterial cytoplasm during adsorption (SE) versus the blocking of phage infections following bacterial entrance into the bacterial cytoplasm (SI). Both occur as a consequence of production of proteins by already infecting phages and both are rather narrowly acting, being limited in their impact especially to closely related phages. In addition, while SE can be displayed by both temperate and nontemperate phages (i.e., phages not able to display lysogenic cycles versus phages that are able to display lysogenic cycles, resp.), SI is limited to just temperate phages. In either case, the result is inactivation of phages that have adsorbed to already phage-infected bacteria. Somewhat equivalently, but using the pharmacokinetic terminology developed above, both SE and SI result in a failure of phage adsorption to activate an adsorbing phage's antibacterial activity (Figure 13).

Lesser known processes can also ensue following phage coinfection such as depressor effects or mutual exclusion, both of which represent reductions in infection burst sizes. While SE and SI are direct and more or less physiologically intended consequences of primary phage gene expression (resulting from intentional protein-phage interactions), the depressor effect and mutual exclusion may be viewed instead as indirect as well as either less- or nonadaptive in terms of phage-phage interactions. Rather, they both are likely consequences of the diverse physiological programs phages display towards successfully modifying their bacterial host and producing phage progeny, resulting therefore in functional incompatibilities between coinciding infections. Multiple adsorptions to the same bacterium also can abort infections including as via lysis from without [170]. These various processes are considered in greater detail elsewhere [99, 191].

Pharmacologically, the dominant impact of these phenomena can be similar to that of multiple phage adsorptions of individual bacteria. That is, generally *n* − 1 phages are inactivated in terms of their bactericidal activity by these processes, where *n* is the number of phages adsorbing to individual bacteria and 1 represents the bactericidal activity of just one of those phages. In light of individual bacteria being able to support the production of only a single phage burst, the net effect of multiple adsorptions per bacterium thus is predominantly a reduction in the efficiency with which phage populations can effect their antibacterial actions. That is, when greater multiplicities of phages are adsorbed to bacteria, particularly when the ratio of adsorbed phages to bacteria comes to exceed one, then the efficiency with which phages kill bacterial targets and amplify their numbers* in situ* can decrease on a per-virion basis.

Recombination also can occur between coinfecting phage genomes, and modification of phage host range is a possible consequence. This can occur due to recombination between two non-temperate phages (such as phages T3 and T7 [192]) or instead phage recombination with a prophage or prophage-like sequence found in the host chromosomes [193]. Genomic studies furthermore reveal substantial gene exchange outside of the laboratory among phages as well as among archaeal viruses [194–196].

## 10. Interaction of Phage-Infected Bacteria with Patient Bodies

The interaction between phage virions and nonmicrobiota aspects of patient bodies, other than in terms of immunological reactions, is thought to be relatively slight, or at least an ongoing aspect of bodies possessing phage-containing normal microbiota [87]. Interaction between phage-infected bacteria and ourselves, on the other hand, can be much less benign. Of prominent concern is the ability of phages—some much more so than others—to transduce genes between bacteria, particularly genes encoding bacterial virulence factors [95, 197–203]. Fortunately, many of these issues can be avoided via informed phage choice, particularly in terms of avoiding temperate phages as antibacterial agents and/or by making sure through bioinformatic analysis that phages both do not and are unlikely to carry bacterial virulence factor genes.

Another issue, phage-mediated release of bacterial lysis products as generated* in situ*, is less easily avoided. Approaches do exist, in terms of both phage modification and design of therapy protocols that can serve to mitigate this concern, some of which are discussed by Goodridge [139]. These approaches include use of lysis deficient phages or limiting rates of phage application to otherwise slow rates of bacterial lysis* in situ*. Alternatively, release of bacterial lysis products is much less of a concern when lysing bacteria are not circulating particularly within blood, just as phage purification need not be as extreme given local versus systemic phage application (above). It is also important to keep in mind that while one of weaknesses of phage therapy is a difficulty in controlling phage population growth under conditions that can support such growth, one of the strengths of phage therapy is that side effects nevertheless tend to be relatively minimal. The most important potential exception to this latter point nonetheless is found with septicemia, particularly with Gram-negative bacteria. Here bacterial lysis can potentially worsen symptoms at least over the short term. The evidence that especially antibiotic-induced bacterial lysis can result in substantially negative clinical outcomes for patients, however, is not robust [204–206].

## 11. Phage Therapy Eco-Physiological Pharmacology

Though leaving out substantial consideration of physiology, the phage therapy writings of Bruce Levin, Rich Lenski, and Jim Bull have long been infused with ecological thinking [47–49, 207–209]. Even earlier, the biocontrol of cyanobacteria literature from especially the 1970s had a strong ecological component; see Abedon [210] for references. In terms especially of mathematical ecology, there has also been consideration, starting at the turn of the current century, in what has been described as “pharmaco-ecology” or “pharmacoecology” [50]. Indeed, as the latter authors suggest (p. 228):

*The concepts explicating the phage-bacteria system have many parallels in theories within ecology and epidemiology that deal with the population dynamics of predator-prey and host-pathogen interactions. It is likely that useful ideas and methodology may be drawn from these areas and perhaps also from experience gained in other forms of biological control. We argue for the incorporation of explicit models of density-dependent replication, to stand alongside knowledge of the relevant physiology and molecular biology if a complete and predictive understanding of phage therapy is to be achieved.*

For additional, ecologically relevant phage therapy articles by this same group, see [52, 188, 211, 212]. See also Weld et al. [213], consideration of the role more generally of ecology in understanding phage therapy [214], and also the general review by Letarov et al. [107] on ecological as well as physiological aspects of phage therapy (see also [65]). The phage therapy literature otherwise has numerous if equivocal references to the “ecology” of various organisms or treated areas of bodies.

Concern about physiology within the context of phage therapy tends to be fairly common, though not often studied in much detail. Issues include those associated with the phage potential to adsorb or otherwise productively infect bacteria such as within the context of biofilms, or phage ability to move from location to location within bodies. See, for example, the work of Levin and Bull [49]. Of relevance as well are issues of secondary pharmacodynamics [64] as well as that of phage absorption such as following* per os* delivery [112].

A primary reason for the relative lack of detailed study of phage physiology or, for that matter, pharmacology within a phage therapy context has to do with the enormous diversity of phages that can be used for phage therapy, with each phage possessing its own, frequently somewhat unique physiology. The often low toxicity of phages, their potential to amplify in number during treatment, and the typically large numbers of different phage types that can be chosen from to treat a given infection furthermore can place a greater premium on issues of phage choice or delivery strategy rather than on the specific physiological or pharmacological underpinnings of phage functionality. Nonetheless, in principle the choice of phage or method of delivery for phage therapy may be improved through better appreciation of the physiology—or indeed multiple physiologies—associated with phage treatment, as well as issues of phage ecology. These issues also may be particularly relevant given phage modification for phage therapy [139, 197] since the properties of unmodified phages at the very least have been tested by natural selection, but that is less true for phage products of biotechnology [215]. Physiological details, however, can be less of a concern to the extent that active phage population growth is not required for therapy success as, for example, one sees with phage-like bacteriocins [168].

The concept of eco-physiological pharmacology—as developed here with regard to phage therapy—considers not just the ecology of a single organism that has been exposed to a bioactive substance but instead is used to characterize a system of organisms of which the individual, “drug”-treated patient is just one component. The system thus contains the host's tissues as well as microbiota, including pathogens, and also the non-self-drug, which in the case of phages also possesses a physiology unto itself. Drugs thus are environmental as well as ecological entities that otherwise are foreign to the body, but bodies themselves also exist as ecosystems. It is within that ecosystem that issues of physiology and pharmacology may be informed by concepts stemming from a large swath of ecological thinking.

## 12. Conclusions

**Figure 14:**
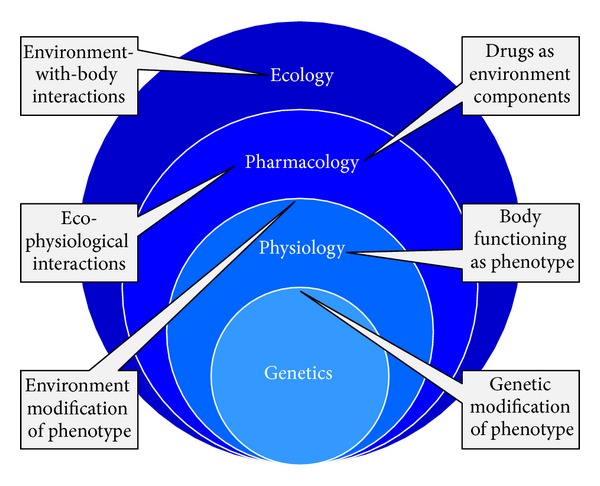
Context of pharmacology as an ecological as well as a physiological phenomenon, with physiology in turn a manifestation of underlying genetics. Pharmacology literally is the exposure of an organism's physiology to an environmental component, that is, a drug, and the study of organism-with-environment interactions literally defines ecology. Similar though arguably less complex “bubbles” can be placed around both bacterial pathogens and their viruses (e.g., Figure 6). Pharmacology thus is inherently eco-physiological while both the pharmaceutical treatment of distinct living entities, such as pathogens, and the use of drugs that themselves are living, particularly bacteriophages, introduces additional aspects of interface between ecology, physiology, and pharmacology.

All entities interact with, are affected by, and in turn impact their environments. If those entities are organisms, then we can label these interactions using ecological terms. If our perspective is from the inside rather than the outside of an organism, then these and other interactions can be viewed instead from the perspective of physiology. If environmental aspects consist of intentionally applied, nonfood, bioactive substances, then it is traditional to consider them instead in terms of their pharmacology (Figure 14), though toxicology as well can be applicable [216]. Overlaps between these different perspectives on organism functioning are substantial. Distinctions are further blurred when a drug's target is itself a distinct organism with its own physiology and ecology, such as a bacterial pathogen, and further still when the “drug” itself is also an organism. The idea of pharmacology as a subset of the study of ecology nevertheless is a more radical proposition than the idea that pharmacology represents, as well, an aspect of the study of physiology. This idea of pharmacology as ecology, as well as physiology, is much less tenably ignored, however, when drugs as well as drug targets themselves both possess a physiology and an ecology.

Here my intention has been to highlight connections that exist between ecology, physiology, and pharmacology, particularly from the perspective of bacterial viruses as living drugs. The goal has not so much been to introduce ecological considerations into pharmacology as to better highlight the parallels between the two disciplines of scientific study, while simultaneously emphasizing the importance of all three biological perspectives to the development of phage therapy. In short and of crucial relevance to the use of phages as drugs, organisms are far more than their genes or genomes but also their phenotypes, and those phenotypes often can be described in both physiological and ecological terms. Organisms as drugs thus may be profitably viewed well beyond their genomics to emphasize as well the far more complex realm of their ecology, physiology, and pharmacology, that is, their eco-physiological pharmacology. Such a viewpoint ultimately represents a more complete perspective on how phages may be employed to combat, within our bodies, especially antibiotic-resistant bacterial pathogens [217, 218] and particularly as antibiotic resistance in bacteria does not tend to also give rise to phage resistance [219, 220].

## Figures and Tables

**Table 1:** Studies involving phage-mediated biocontrol and therapy of bacteria from approximately the first six months of 2013.

Target	Context	Classification^*^	References
*Clostridium difficile *	*In vitro* colon model	Therapy	[228]
*Escherichia coli *	Beef	Biocontrol	[229]
*Escherichia coli *	Chickens (colibacillosis)	Therapy	[230]
*Escherichia coli *	Milk (during fermentation)	Biocontrol	[231]
*Escherichia coli *	Beef	Biocontrol	[232]
*Escherichia coli *	Vegetables	Biocontrol	[233, 234]
*Mycobacterium ulcerans *	Mouse footpad model	Therapy	[235]
*Pectobacterium carotovorum *	Lettuce	Both	[236]
*Pseudomonas aeruginosa *	*In vitro* and tooth biofilm models	Therapy	[237]
*Pseudomonas aeruginosa *	*In vitro* biofilm model	Biocontrol	[238, 239]
*Salmonella enterica *	Chicken skin (food)	Biocontrol	[240]
*Salmonella enterica *	Eggs	Biocontrol	[241]
*Salmonella enterica *	Various foods	Biocontrol	[242]
*Salmonella gallinarum *	Chickens	Therapy	[243]
*Shigella* spp.	Chicken (food)	Biocontrol	[244]
*Staphylococcus aureus *	Rabbit wound model	Therapy	[151]
*Staphylococcus aureus *	Mouse diabetic foot model	Therapy	[245]
*Staphylococcus aureus *	Rat implant model	Therapy	[223]
*Vibrio cholerae *	Rabbit model	Therapy	[246]
*Vibrio coralliilyticus *	Coral	Both	[247]

*Classification is based on the discussion of Abedon [78]. Generally, “therapy” involves treatment of *disease* in infected or potentially infected *individuals*; that is, therapy thus is both of infectious, bacteria-caused disease and of an individual disease-carrying host. “Biocontrol” by contrast involves treatment of environments, variously defined. Therapy also can be viewed as phage use as a drug or especially antibiotic equivalent whereas biocontrol, narrowly defined, is phage use as a disinfectant or antiseptic equivalent. “Both” refers to treatments that could be classified as either biocontrol or therapy because individuals are being treated within the context of the treatment of environments such as the use of phages in agriculture against plant pathogens. In either case, treatment versus prevention is not distinguished in the table.

**Table 2:** Studies involving phage-mediated biocontrol and therapy of bacteria from 2012.

Target	Context	Classification^*^	References
*Acinetobacter baumannii *	*In vitro* biofilm model	Therapy	[248]
*Bacillus cereus *	*Cheonggukjang* (food)	Biocontrol	[125]
*Campylobacter* spp.	Meat (chicken, pork)	Biocontrol	[249]
*Dickeya dianthicola *	Potato soft rot	Both	[250]
*Escherichia coli *	Chickens (diarrhea)	Therapy	[251]
*Escherichia coli *	*In vitro* biofilm model	Therapy	[252, 253]
*Escherichia coli *	Mouse intestinal model	Therapy	[254]
*Escherichia coli *	Mouse model	Therapy	[122]
*Escherichia coli *	Rat pup model	Therapy	[255]
*Escherichia coli *	Lettuce/Beef	Biocontrol	[256]
*Gordonia* spp.	Activated sludge foaming model	Biocontrol	[257]
*Klebsiella pneumoniae *	Mouse model	Therapy	[258]
*Listeria monocytogenes *	Cheese	Biocontrol	[259]
*Pseudomonas aeruginosa *	*Ex vivo* human skin	Therapy	[260]
*Pseudomonas aeruginosa *	Mouse keratitis model	Therapy	[261]
*Pseudomonas aeruginosa *	Mouse lung infection model	Therapy	[262]
*Pseudomonas aeruginosa *	Wax moth larvae model	Therapy	[263]
*Ralstonia solanacearum *	Tomato bacterial wilt	Both	[264, 265]
*Salmonella enterica *	Chickens	Biocontrol	[266, 267]
*Salmonella enterica *	Mouse model	Therapy	[267]
*Salmonella enterica *	Various foods	Biocontrol	[268]
*Staphylococcus aureus *	Cheese	Biocontrol	[269]
*Staphylococcus aureus *	*In vitro* biofilm model	Therapy	[270]
*Vibrio coralliilyticus *	Coral	Both	[271]
*Yersinia enterocolitica *	Meat (chicken, pork)	Biocontrol	[249]
*Yersinia pestis *	Mouse model	Therapy	[272]

*See equivalent footnote for Table 1.
